# Enhancing resistant starch and quality of mung bean starch and glass noodles through optimized incubation times and temperatures

**DOI:** 10.1016/j.fochx.2025.103111

**Published:** 2025-10-01

**Authors:** Ratchaneeporn Photinam, Pakkawat Detchewa, Anuchita Moongngarm

**Affiliations:** a Agriculture and Food Technology section, Nuclear Technology Research and Development Center, Thailand Institute of Nuclear Technology, Nakhon Nayok 26120, Thailand; b Department of Food Science, Faculty of Science, Burapha University, Chonburi 20131, Thailand; c Department of Food Technology and Nutrition, Faculty of Technology, Mahasarakham University, Maha Sarakham 44150, Thailand

**Keywords:** Freezing, Gluten-free noodle, Mung bean, Optimization, Resistant starch, Starch digestibility, XRD crystallinity

## Abstract

This study investigated the effects of incubation temperatures (25 °C, 4 °C, and −10 °C for 48 h) on the physicochemical properties and starch digestibility profiles of autoclaved mung bean starch. The most effective condition (−10 °C) was applied for glass noodle production. The noodles were processed under varying freezing times (19–53 h) and cold incubation (0–9.3 h), following a central composite design (CCD) and analyzed using response surface methodology (RSM). Incubation at −10 °C promoted a C to B-type crystallinity shift, yielding the highest resistant starch (RS) (15.85 %). Freezing time had a stronger influence than cold incubation on RS enhancement in a real food matrix, with optimal RS obtained after 19–25 h of freezing and less than 5 h of cold incubation. Integrating structural analysis and process optimization provided a practical pathway to develop RS-enriched, low-glycemic mung bean glass noodles for health-conscious consumers.

## Highlights

• Maximum RS content of autoclaved mung bean starch was observed at −10 °C.
• A Shift from C to B-type crystallinity was found in mung bean starch retrogradation.
• This study optimized RS in mung bean glass noodles via freezing–incubation.
• Freezing time impacted RS yield in glass noodles more than cold incubation.
• Optimal RS content was achieved under 19–25 h freezing and <5 h cold incubation.

## Graphical abstract

**Figure:**
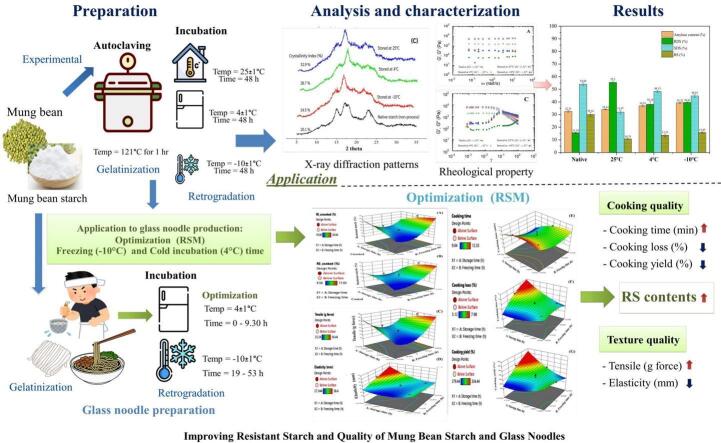

## 1 Introduction

Mung bean (*Vigna radiata* L.) is a globally cultivated edible legume. Its seeds are a rich source of carbohydrates, vitamins, minerals, fiber, and protein, with an ideal essential amino acid profile, while its starch is suitable for producing glass noodles as it contains high amylose and resistant starch (RS), which contribute to desirable gel firmness, elasticity, and resistance to retrogradation (Kabas et al., 2007). RS does not digest in the small intestine and is fermented in the colon, offering health benefits similar to dietary fiber and prebiotics, including improved digestive health and modulation of blood glucose. Increasing the RS content in staple foods is a key nutritional goal, particularly for the development of low-glycemic functional foods (Raigond et al., 2015). Heat treatment, especially autoclaving followed by cold storage, enhances mung bean starch functionality by increasing RS through retrogradation and changes in crystalline structure (Raungrusmee et al., 2022). Mung bean starch requires heat treatment to enhance its functional characteristics and expand its applicability across various industries. Native starches often exhibit limitations such as poor solubility, instability under extreme conditions, and uncontrolled digestibility, restricting their practical use. Physical treatments such as temperature, moisture, pressure, shear, and irradiation are simple, safe, and effective methods for modifying starch properties. Heat treatment, especially autoclaving followed by low-temperature incubation, promotes starch retrogradation, alters the crystalline structure, and increases the RS content (Raungrusmee et al., 2022). These modifications reorganize starch granule molecular arrangements, affecting physicochemical properties, rheology, and morphology. RS, an important starch fraction formed during this modification, enhances digestibility control and contributes to functional food development with lower glycemic indices. Heat treatment also improves starch gelatinization, retrogradation, and pasting behavior, enhancing its role as a thickening or stabilizing agent and increasing resistance to harsh processing conditions such as freezing, drying, and acidic environments.

Structural changes induced by heat include disruption of the semi-crystalline granule structure, partial gelatinization, and amylose leaching, which influence viscosity, water absorption, and gel stability. Post-heat incubation conditions further modulate starch properties. Low-temperature incubation promotes retrogradation and RS formation, improving gel stability and making starch ideal for health-oriented foods, whereas high-temperature incubation accelerates gelatinization and increases rapidly digestible starch (RDS), but prolonged exposure can reduce RS levels. Autoclaving at or above 120 °C followed by cooling increases RS and amylose yields (Raungrusmee et al., 2022), while RS content significantly increases when cooked starch is cooled to sub-zero temperatures (−20 °C) (Kim et al., 2006). Viscoelasticity measurements provide insight into the relationship between starch microstructure and rheological behavior, further linking processing conditions to functional properties (Ettehadi et al., 2022). Following heat treatment, incubation conditions critically influence both RS formation and functional properties of mung bean starch. Low-temperature incubation enhances retrogradation, promoting RS formation, improving gel stability, and maintaining desirable viscoelastic properties, which are essential for functional and health-oriented foods. By contrast, higher incubation temperatures accelerate gelatinization and increase RDS, but prolonged exposure may reduce RS content. These mechanistic insights provide a foundation for translating starch modification into practical food applications.

Glass noodles or cellophane noodles, primarily produced from starches such as mung bean, yam or cassava, are a staple food in many Asian cuisines (Gao et al., 2024). Mung bean starch is particularly valued for its elevated amylose content, which promotes the formation of a strong, continuous gel network and results in noodles with superior firmness, elasticity, and reduced cooking loss. High amylose content also enhances resistance to retrogradation by limiting syneresis and hardening upon cooling, thereby contributing to a desirable texture during refrigerated storage and extending shelf-life. Functional attributes such as gel strength and thermal and mechanical stability contribute to producing high-quality noodles. Processing techniques, including freezing (−18 °C to −5 °C) and cold incubation, improve noodle texture by increasing RS content, enhancing chewiness, and reducing stickiness (Nguyen et al., 2023). Subsequent aging at 4 °C for 21 h stabilizes the starch gel, increasing hardness and elasticity (Tang et al., 2019). Mung bean starch exhibits favorable water-binding capacity and low cooking loss, properties essential for consumer acceptability and industrial efficiency (Liu et al., 2025). Controlled hydrothermal treatments, such as partial gelatinization at 70 °C, modify the starch structure by reducing the crystallinity and molecular order, thereby influencing digestibility and glycemic response (Awais et al., 2020). Recent studies indicated that targeted physical modifications enhanced functional and nutritional properties. Heat-moisture treatment (HMT) at 120 °C significantly increased RS levels in mung bean starch, along with higher apparent amylose content and changes in microstructure and crystallinity. Similarly, ultra-high-pressure (UHP) gelatinization followed by prolonged storage induced regenerated C-type crystallinity and a gradual increase in RS content, accompanied by alterations in swelling power, solubility, and thermal stability (Li et al., 2015). Evidence from other legume and cereal systems also suggests that autoclaving followed by cooling boosts RS formation through molecular compaction Arroqui et al. (2025), with dual treatments showing promise for mung bean starch applications. Detailed structural characterization revealed that RS was reduced after cooking, but a substantial fraction remained as slowly digestible starch (Yao et al., 2019), highlighting the importance of optimizing processing strategies to enhance RS for improved nutritional quality. Despite these advances, the combined influence of autoclaving and low-temperature storage on RS formation, starch crystallinity, and the functional properties of mung bean starch in glass noodle production remains underexplored and warrants further investigation. Extensive research has addressed mung bean starch functionality and RS enhancement, but the combined influence of autoclaving and low-temperature storage on RS formation, starch crystallinity, and functional properties remains underexplored. To address this gap, the present study was designed in two integrated stages. The objectives of this study were to systematically investigate the effects of incubation temperature (25 °C, 4 °C, and −10 °C) on RS formation, crystallinity, and physicochemical properties of autoclaved mung bean starch under controlled laboratory conditions. The optimal condition was then employed (−10 °C) for glass noodle production, using response surface methodology (RSM) to optimize freezing and cold incubation durations for maximum RS enrichment and desirable textural properties. This stepwise approach connected fundamental starch modification to practical product development, providing both mechanistic understanding and application guidance for RS-enriched, low-glycemic foods.

## 2 Materials and methods

### 2.1 Mung bean starch modification using heat treatment

Mung bean starch was prepared and modified following the method of Photinam et al. (2016) with some modifications to investigate the effect of incubation temperatures on starch digestible profiles and physicochemical properties. A moisture content of 30 % was selected to ensure adequate water availability for starch gelatinization during autoclaving, as moisture levels ranging from 20 % to 40 % effectively promoted gelatinization and retrogradation in starch-based systems (Photinam et al., 2016). A 30 % moisture level facilitated the molecular mobility necessary for the structural rearrangements leading to RS formation. Mung bean (*Vigna radiata* L. R. Wilcz) seeds were purchased from a local market in Mahasarakham, Thailand. Ten grams of mung bean starch were prepared by adjusting the moisture content to 30 % with distilled water. The suspension was then subjected to autoclaving at 121 °C for 1 h. After autoclaving, the starch paste was allowed to cool to room temperature for 1 h and subsequently stored at 25, 4, and −10 °C for 48 h. Following incubation, the starch pastes were dried in an oven at 45 °C for 12 h to reach a consistent moisture content of 10 %. The dried starch was then finely ground, passed through a 70-mesh sieve, and stored in sealed bags at room temperature until further analysis.

#### 2.1.1 Determination of amylose content

The amylose content was determined following the modified method of Photinam and Moongngarm (2023). A 100 mg starch sample was dissolved in 1.0 mL of 95 % ethanol, mixed with 9 mL of 1 M NaOH, and heated for 20 min. After cooling, the solution was diluted and treated with I₂/KI solution and acetic acid. The absorbance was measured at 620 nm using a Shimadzu UV-1800 Spectrophotometer, Shimadzu, Japan. The amylose concentration was calculated from a standard curve (Fig. S) prepared using potato amylose (Sigma-Aldrich, USA) at concentrations ranging from 0 to 100 mg/mL. Each sample was analyzed in triplicate, with amylose content expressed as a percentage of total starch.

#### 2.1.2 Determination of RS, SDS and RDS

Rapidly digestible starch (RDS), slowly digestible starch (SDS), and total RS contents were determined using a Megazyme Digestible and Resistant Starch Assay Kit (K-DSTRS) (Megazyme, Bray, Ireland) as Englyst et al. (1996), reflecting the in vivo starch digestion rate.

#### 2.1.3 Pasting properties

The pasting properties of a 6 % (*w*/w) starch dispersion were determined using a rapid viscosity analyzer (Newport Scientific, Warriewood, New South Wales, Australia), following the method of Photinam and Moongngarm (2023).

#### 2.1.4 Swelling power and solubility index

Heating temperatures were selected for the swelling power and water solubility index (WSI) analyses based on the established literature (Photinam et al., 2016). The range 55–95 °C spanned temperatures below, near, and above the gelatinization temperature of mung bean starch (65–75 °C), enabling a comprehensive assessment of starch behavior throughout its gelatinization profile. The starch (0.1 g) was mixed with 10 mL of water and heated at varying temperatures (55–95 °C). WSI and SP were calculated using weight measurements before and after drying.

#### 2.1.5 X-ray diffraction (XRD)

XRD patterns of starch were obtained using the TTRAX III system (Rigaku, Japan) with CuK radiation (λ = 0.1542 nm), following the method of Photinam and Moongngarm (2023). The analysis was performed in the 2θ range of 4°–35°, using a step width of 0.04° and a count time of 1 s per step at 30 mA and 40 kV. Relative crystallinity was calculated using the equation:

(1) $$\mathrm{Relative crystallinity}\;\left(\%\right)=\mathrm{Ac}/\left(\mathrm{Ac}+\mathrm{Aa}\right)$$

where Ac is the crystallized area on the X-ray diffractogram, and Aa is the amorphous area on the X-ray diffractogram.

#### 2.1.6 Rheological property measurement

Starch samples (15 % *w*/w) were heated at 95 °C while stirring for 15 min. A 0.5 mm thick plate sample was formed and maintained at 60 °C for 5 min. Dynamic viscoelastic properties were evaluated using a rotational rheometer (Rheology Co., Ltd., MR-300 V2E) and fixtures of parallel plates (electrodes) made of stainless steel at 20 °C. A strain amplitude of 1 % was applied with a frequency of 1.5 Hz in the linear viscoelastic region.

### 2.2 Application of optimal incubation conditions to prepare glass noodles

The optimal condition from Part 1 (−10 °C) was applied to investigate the effects of freezing and cold incubation on the RS content and quality of mung bean glass noodle production, a complex matrix requiring parameter adjustments. RSM methodology was used to optimize the freezing duration and subsequent cold incubation to maximize RS content while maintaining desirable texture. Part 1 findings showed that −10 °C most effectively promoted RS₃ formation compared with 4 °C and 25 °C, facilitating optimal molecular rearrangements during retrogradation while minimizing ice crystal damage. Consequently, −10 °C was used in Part 2 to refine freezing and cold incubation times for glass noodle production, ensuring that processing was conducted at the most favorable temperature.

#### 2.2.1 Response surface methodology for the preparation of glass noodles

A central composite design (CCD) with freezing times at −10 °C ranging from 19.00 to 53.00 h and cold incubation times at 4 °C from 0.00 to 9.30 h was employed, using response surface methodology (RSM) to optimize the preparation conditions of glass noodles. The experimental design included 13 treatments, comprising one axial point and four core points. The experiment was replicated three times, utilizing five vertices and one central point to assess the characteristics of the noodles, with results analyzed by Design-Expert® v.12.00 (Stat-Ease, Minneapolis, MN, USA).

Incubation at 4 °C was set at 0.00, 2.00, 5.00, 8.00, and 9.30 h, while freezing at −10 °C was established at 19.00, 24.00, 36.00, 48.00, and 53.00 h. An empirical quadratic polynomial model was fitted to the response variable (Y) using the equation:

(2) $$Y=\beta_{o}+\sum_{i=1}^{2}\beta_{i}X_{i}+\sum_{i=1}^{2}\beta_{\mathrm{ii}}X_{i}^{2}+\sum_{i=1\;j\neq 1}^{2}\beta_{\mathrm{ij}}X_{i}X_{J}$$

where Y is the dependent or response variable, and *βo*, *βi*, *βii*, and *βij* are the constants, linear, quadratic, and cross-product regression coefficients, respectively. The coded independent variables *X1* and *X2* were referred to as *Xi*. ANOVA was conducted to analyze the results at a significance level of *P* = 0.05. The model's adequacy was assessed using the coefficient of determination (R^2^) and the model's *P*-value. This approach facilitated a thorough evaluation of the factors affecting glass noodle quality.

#### 2.2.2 Glass noodles preparation

The preparation of glass noodles followed the method outlined by Photinam et al. (2016), with slight modifications. A mixture was created using 950 g of raw mung bean starch and 50 g of cooked starch, totaling 1 kg of starch blend. To produce cooked starch (pre-gelatinized starch), 50 g of raw starch was combined with 250 mL of water and heated for 5 min. The cooked starch was then mixed with additional water to create a starch slurry. The dough was adjusted to a moisture content of 50 %, and extruded using a hand-operated mincer into a 0.5 cm diameter stainless-steel cylinder. The extruded noodles were immediately dropped into boiling water (98–100 °C) and cooked for 3 min in a rhythmic, continuous flow. The noodles floated to the surface when fully cooked. After cooking, the glass noodles were rinsed in cold water and drained before storing under two conditions as refrigerated at 4 °C for 0–9.3 h and frozen at −10 °C for 19–53 h. Finally, the noodles were oven-dried at 45 °C for 8–12 h until reaching a final moisture content of 10–14 %. Once dried, the glass noodles were stored at room temperature in polyethylene bags.

#### 2.2.3 Determination of cooking quality

Cooking time, cooking yield, and cooking loss were determined following (Chemists, 2000). The cooking yield of the cooked glass noodle samples was calculated using 10 g (dry weight) boiled in 300 mL of water, followed by washing with 50 mL of distilled water, draining for 5 min, and immediately weighing to measure the percentage increase in weight:

(3) $$\mathrm{Cooking yield}\;\left(\%\right)=\frac{M1-M2\times \left(1-W\right)\;}{M2\times \left(1-W\right)}\times 100$$

where *M1* is the weight of the glass noodles (g) after cooking, *M2* is the weight (g) of the glass noodles (before cooking), and *W* is the moisture content (%) of the glass noodles (before cooking). To calculate the cooking loss, the cooking water was collected, placed in a moisture container, and dried at 105 °C to a consistent weight. The glass noodle cooking loss was determined using the equation:

(4) $$\mathrm{Cooking loss}\;\left(\%\right)=\frac{M}{G\times \left(1-W\right)}\times 100$$

where G is the weight of the glass noodles before cooking, W is the moisture content before cooking, and M is the weight of the dried glass noodles (dry matter) (g).

#### 2.2.4 Textural analysis

The tensile strength of the mung bean glass noodles was measured using a texture analyzer (TA-XT2, Stable Micro Systems, Surrey, UK), following the method of Ritthiruangdej et al. (2011). The noodles were boiled, cooled, and arranged in a straight line. The analyzer was set at pre-test speed 3.0 mm/s, post-test speed 5.0 mm/s, test speed 3.0 mm/s, trigger distance 100 mm, and auto 5.0 g. Tensile strength (maximum force; g) and breaking length (distance at maximum force; mm) were calculated from the force-distance curves.

### 2.3 Statistical analysis

The experimental data were analyzed using analysis of variance (ANOVA) and presented as mean values with standard deviations. Duncan's multiple range test was applied to identify significant differences between means at a significance level of *P* < 0.05. Statistical analyses were performed using SPSS version 23.0 for Windows. Experimental design and data evaluation were conducted using Design Expert (version 12.0.0, Stat-Ease, Inc., Minneapolis, MN). Pearson's correlation coefficients were calculated to assess the relationships between various parameters, with a significance threshold set at *P* ≤ 0.05, indicating statistically significant associations among the measured attributes.

## 3 Results and discussions

### 3.1 Effect of incubation temperature on starch digestible profiles and physicochemical properties of mung bean starch

#### 3.1.1 Amylose content and starch digestibility profiles

The amylose content in native mung bean starch was 32.51 %. Autoclaved treatments stored for 48 h at lower temperatures demonstrated a significant increase in amylose content, ranging from 34.14 to 39.32 %. The highest amylose content (39.32 %) was observed at −10 °C, as shown in Table 1. This increase in amylose content was associated with a rise in RS levels, as the amylose molecules aligned and formed links during cooking and cooling. In terms of starch digestibility, native mung bean starch contained 10.58 % RDS, 48.16 % SDS, and 41.16 % RS. After autoclaving and incubation at 25 °C, 4 °C, and −10 °C, the RS content decreased, while RDS and SDS levels significantly increased. The highest RS content (15.85 %) at −10 °C was followed by 4 °C (13.53 %) and 25 °C (10.74 %), while the RS content in autoclaved starch without incubation markedly reduced from 30.16 % in native starch to 8.23 % (data not shown). This finding concurred with Ma et al. (2017), who reported that the RS content of cooked starch (9 %) was significantly lower than raw starch (56 %). These results suggested that lower incubation temperatures favored the formation of a double helix structure, contributing to the formation of RS3. Lower temperatures enhanced starch retrogradation by slowing molecular motion, allowing the amylose and amylopectin molecules to realign into a more ordered, crystalline structure, leading to stable double helices and increased RS3 content. Thermodynamic factors at low temperatures also strengthened hydrogen bonding between the hydroxyl groups in the starch chains, stabilizing the granules and making them more resistant to enzymatic digestion. Water migration also played a key role, as cooling facilitated the gradual removal of water from the starch chains, promoting stronger intra and intermolecular interactions that enhanced crystallinity and RS3 formation. Lower storage temperatures promoted a shift from C-type to B-type crystallinity, as evidenced by the XRD patterns, showing the disappearance of the characteristic C-type peak at 17.5 and 18.0° (2θ) and the emergence of B-type peaks at 6.0°, 17.0°, and 22.0° (2θ). The B-type crystallinity was more resistant to enzymatic hydrolysis and closely associated with increased RS3 levels, consistent with studies reporting the highest RS content after storage at −10 °C (Li et al., 2022). These changes confirmed the structural transformation of the starch crystalline pattern following treatment. The optimization of incubation temperatures facilitated starch retrogradation and double helix formation, ultimately increasing the RS3 content and enhancing the functional properties of starch-based foods. These findings were supported by Li et al. (2024).

**Table 1:** Amylose contents, starch fractions and pasting properties of native mung bean and autoclaved treatments of mung bean starch after incubation at 25 °C, 4 °C, and −10 °C for 48 h.

Starch/Condition	Amylose content (%)	Starch fractions (%)			Viscosity (RVU)						
		RDS	SDS	RS	Peak (RVU)	Breakdown (RVU)	Trough (RVU)	Setback (RVU)	Final (RVU)	Peak time (min)	Pasting temp (°C)
Native	32.51 ± 0.72^d^	15.58 ± 0.40^c^	54.04 ± 1.26^a^	30.16 ± 1.36^a^	1083.89 ± 3.66^a^	562.92 ± 1.87^a^	464.03 ± 2.50^a^	255.80 ± 1.23^d^	665.27 ± 0.57^c^	3.82 ± 0.04^e^	74.08 ± 0.92^a^
25 °C	34.14 ± 0.35^c^	55.50 ± 1.09^a^	31.97 ± 1.50^d^	10.74 ± 0.51^d^	457.95 ± 1.01^b^	235.54 ± 1.94^c^	220.03 ± 5.60^b^	400.68 ± 2.32^b^	1006.81 ± 2.46^a^	4.97 ± 0.24^b^	67.51 ± 0.49^b^
4 °C	36.93 ± 0.64^b^	38.14 ± 2.29^b^	48.33 ± 1.00^b^	13.53 ± 1.14^c^	430.49 ± 6.37^c^	181.12 ± 6.26^d^	172.32 ± 5.87^c^	356.50 ± 0.72^c^	580.24 ± 6.98^d^	5.15 ± 0.02^ab^	65.36 ± 0.80^c^
−10 °C	39.32 ± 0.44^a^	39.61 ± 0.47^b^	44.81 ± 1.11^c^	15.85 ± 0.61^b^	436.32 ± 5.11^c^	279.29 ± 4.00^b^	163.29 ± 1.88^d^	505.06 ± 3.93^a^	739.97 ± 5.09^b^	4.45 ± 0.08^cd^	63.07 ± 0.53^c^

Results are mean ± standard deviation of triplicate determinations. Different letters in the same columns indicate significant differences (*P* < 0.05).

#### 3.1.2 Pasting properties

The pasting temperature of native mung bean starch was 74.08 °C, higher than the autoclaved starch treatments stored at 25 °C, 4 °C, and −10 °C, with pasting temperatures ranging from 63.07 °C to 67.51 °C, as shown in Table 1 The decrease in pasting temperature from native starch to the −10 °C treatment (63.07 °C) indicated increased susceptibility to gelatinization after autoclaving and cold storage. Peak time increased in autoclaved starches, especially at 4 °C (5.15 min) and 25 °C (4.97 min) versus native starch (3.82 min), indicating delayed gelatinization from greater molecular ordering. Autoclaved mung bean starch stored at low temperatures exhibited longer peak times (4.45–5.15 min) compared to native starch (3.82 min). Starch samples stored at 25 °C, 4 °C, and −10 °C had higher pasting temperatures and prolonged peak times, resulting in lower peak viscosity, setback, and final viscosity compared to the native starch. The increase in peak time suggested that the starch structure was strengthened, indicating enhanced intramolecular bonding. Yi et al. (2021) highlighted a significant correlation between RS content and RVA parameters, explaining the observed reductions in paste viscosity, except for peak time, due to structural changes in autoclaved starch. Results suggested that the starches were less prone to swelling and gelatinization, aligning with the formation of a more compact and less digestible structure. Setback viscosity, an indicator of retrogradation, was lowest in native starch (255.80 RVU) and highest after storage at −10 °C (505.06 RVU), indicating greater retrogradation at low temperature. Yuan et al. (2007) noted that the lower setback values observed in autoclaved starch during cooling and incubation were attributed to the larger granule size, making the starch more fragile as its diameter increased. Other pasting parameters including breakdown, trough, setback, and final viscosity varied significantly across treatments, indicating structural changes and altered retrogradation behavior.

#### 3.1.3 Swelling power and solubility index of mung bean starch

**Fig. 1:**
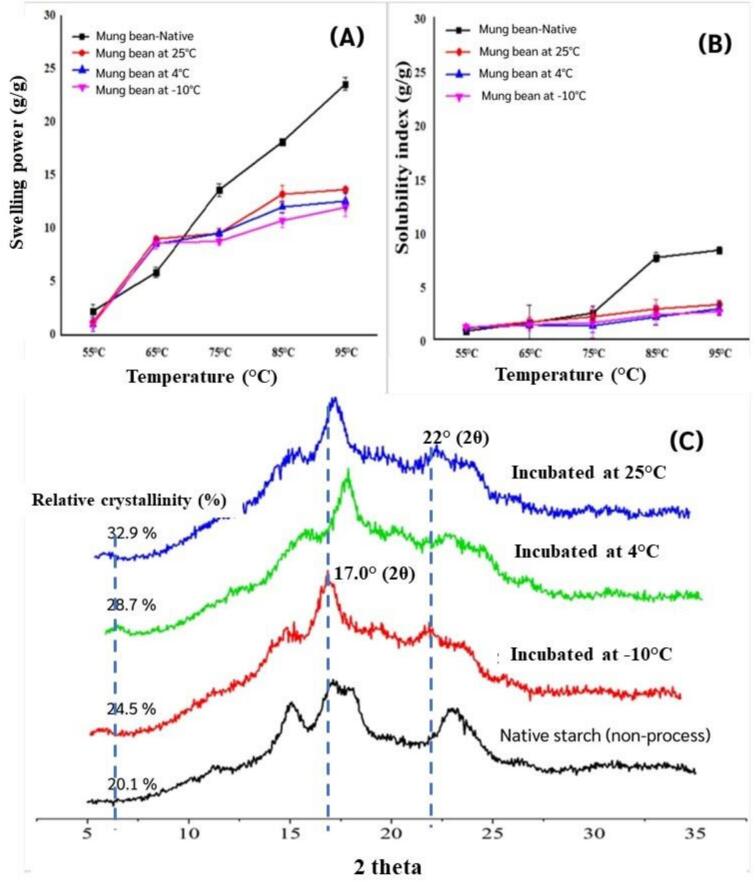
(A) Swelling power, (B) Solubility index, and (C) X-ray diffraction patterns of mung bean native starch (non-processed), autoclaved treatments after incubation at 25 °C, 4 °C, and −10 °C for 48 h, respectively.

The swelling power of native mung bean starch increased significantly with temperature, ranging from 55 to 95 °C (Fig. 1A). Native starch exhibited higher swelling power compared to autoclaved starches stored at lower temperatures. As the temperature increased, swelling also increased, with significant differences observed. At 95 °C, native starch reached its peak swelling power, while autoclaved starches stored at lower temperatures showed reduced swelling capacity, with values ranging from 11 to 13 g/g.

Solubility, linked to the presence of soluble molecules like amylose, reflects the ability of solids to dissolve in water. Native starch demonstrated low solubility at 55 °C and 75 °C because these temperatures were below its gelatinization point. Solubility began to rise after 75 °C, with a significant increase in the solubility index as the temperature continued to climb (Fig. 1B). The solubility index of native starch remained below 10 %, consistent with Zhang et al. (2019), who reported that solubility in legumes increased rapidly after 70 °C due to the disorganization of starch granules near their gelatinization temperature. By contrast, autoclaved starches stored at lower temperatures exhibited limited solubility, with values ranging from 2.5 to 3.5 g/g. This decrease was attributed to amylose leaching during autoclaving, which altered the distribution of soluble solids within the swollen starch granules (Mathobo et al., 2021). During gelatinization, solubilized amylose diffused out of the granules and, upon cooling, reassociated to form double helices and crystalline regions. These structures promoted retrogradation, strengthening the gel network and increasing RS3 content by enhancing resistance to enzymatic hydrolysis. Greater amylose leaching is generally associated with firmer gel texture and higher retrograded starch levels. The extent of leaching depends on starch type and processing conditions; excessive losses (15–20 %) can reduce RS3 formation and negatively affect cooking quality and texture (Feng et al., 2024).

#### 3.1.4 X-ray diffraction patterns of mung bean starch

The X-ray diffraction (XRD) patterns of native mung bean starch exhibited distinct peaks at 15.0°, 17.0°, 17.5°, 18.0°, and 23.2° (2θ), characteristic of a C-type crystalline structure as a mixture of both A- and B-type crystallinities, as shown in Fig. 1C. Native starch had a relative crystallinity of ∼20.1 %. Autoclaved mung bean starch stored at 25 °C, 4 °C, and −10 °C for 48 h showed typical B-type crystalline peaks, with relative crystallinity increasing to 24.5 %, 28.7 %, and 32.9 %, respectively, indicating enhanced crystallinity after treatment and incubation. By contrast, autoclaved mung bean starches stored at 25 °C, 4 °C, and −10 °C for 48 h displayed a strong diffraction peak at 17.0° along with weaker peaks at 5.5°, 15.0°, 16.3°, 17.1°, and 23.2°, indicative of a typical B-type crystalline structure. The B-type crystallinity is marked by a well-defined peak at 17°, corresponding to the aggregation state of amylose double helices (Chen et al., 2019). The shift from C-type to B-type crystallinity indicates amylose recrystallization rather than amylopectin retrogradation. This process strengthens starch granules and amylose structure, increasing gel firmness and relative crystallinity (Mathobo et al., 2021). The transition from C-type to B-type crystals was associated with a reduction in starch digestibility, due to the formation of resistant starch (RS3). The most significant crystallinity was observed in samples stored at −10 °C, indicated by the most intense and well-defined peaks. Freezing at this temperature significantly promoted starch retrogradation, due to the restricted molecular mobility of water, facilitating the formation of stable crystalline regions. These findings concurred with Nguyen et al. (2023), underscoring the significant influence of incubation temperature on the retrogradation and crystallinity of mung bean starch, with lower temperatures (−10 °C and 4 °C) promoting higher crystallinity, and incubation at 25 °C resulting in comparatively lower crystallinity. These results highlighted the importance of temperature control in managing the texture and shelf life stability of starch-based food products.

#### 3.1.5 Dynamic viscosity of mung bean starch

**Fig. 2:**
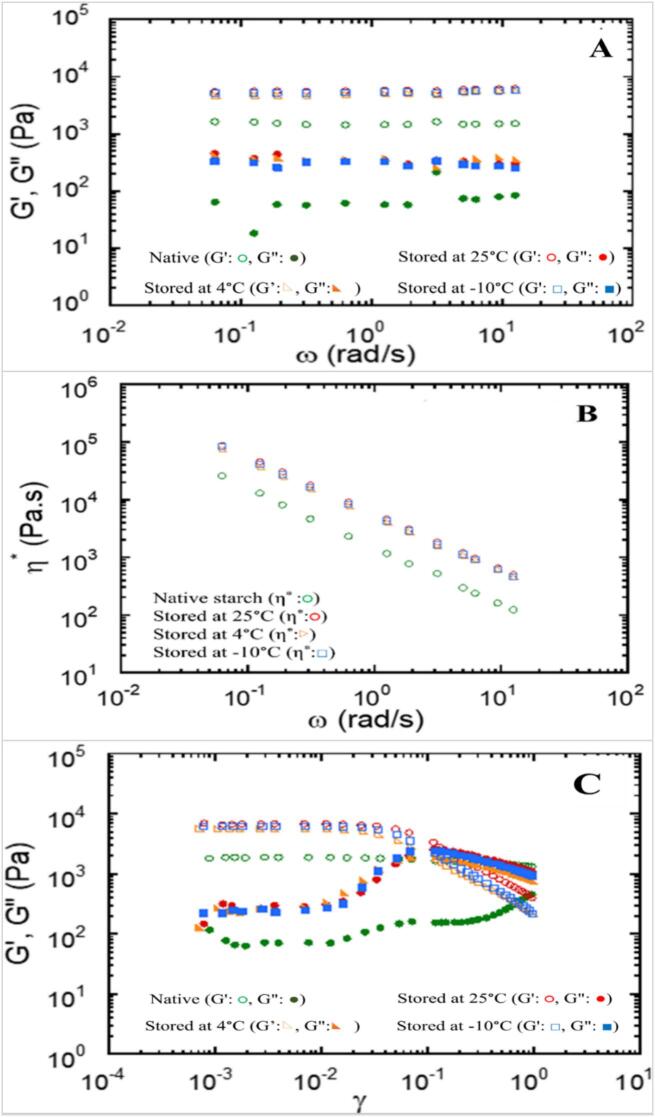
(A) Storage moduli (G′) and loss moduli (G″) plotted against frequency (ω), (B) Complex viscosity (η*) as a function of frequency (ω), and (C) Storage (G′) and loss (G″) modulus dependence on oscillatory strain (γ) for aqueous solutions of mung bean retrogradation treatments stored at 25 °C, 4 °C, and −10 °C compared with non-processed native starch.

Frequency dependencies: the viscoelastic behavior of autoclaved mung bean starch, stored at three different temperatures, was analyzed across a frequency range of 0.1–10 rad/s, in comparison to native starch (Fig. 2A). The autoclaved starch consistently exhibited higher incubation modulus (G′) values than loss modulus (G″) throughout the frequency range, indicating a gel-like structure with enhanced gel strength and high setback viscosity. Previous studies noted that freeze-thaw cycles at −20 °C increased incubation moduli, resulting in firmer gels during frequency sweeps (Vernon-Carter et al., 2016). Higher amylose content led to a more solid-like paste, as reported by Irani et al. (2019), and influenced gel behavior. Rheological analysis revealed that the incubation modulus (G′) consistently exceeded the loss modulus (G″) for all incubation temperatures, suggesting predominantly gel-like properties in the starch slurries. This gel structure remained largely frequency-independent, indicating a stable network that could be beneficial in food applications requiring thickening or gelling properties. Amylose reassociation during cooling forms a gel network that directly defines noodle texture. Higher G′ values are associated with firmer, more elastic, and less sticky noodles, whereas lower values result in softer and stickier textures. Thus, the viscoelastic properties of starch can be used to predict and explain the textural quality of glass noodles.

Complex viscosity: both autoclaved and native starches exhibited non-Newtonian shear-thinning flow behavior (Fig. 2B). Complex viscosity (η*) decreased linearly with increasing frequency, confirming reduced viscosity in response to shear rate and time. This behavior suggested that autoclaved mung bean starch had superior thickening and stabilizing potential in food applications compared to native starch (Hesarinejad et al., 2018). Non-Newtonian, shear-thinning behavior is typical in food systems such as noodle dough. Complex viscosity reflects the resistance to deformation by integrating both viscous and elastic responses of the material. High viscosity at low frequencies indicates a strong structural network, which is beneficial for cooking stability and handling. However, excessively high viscosity can impede processing flow, highlighting the importance of balancing structural strength with processability in practical noodle production.

Strain amplitude dependencies: the strain amplitude behavior of autoclaved starch, stored at various temperatures, showed that at low deformation levels, G′ remained relatively constant and greater than G″, indicative of gel-like properties (Fig. 2C). When the strain exceeded a critical threshold (γ = 0.05), G′ gradually declined, signaling the failure of the internal gel structure and a change to dominant viscous behavior. This behavior aligned with Torres et al. (2017). By contrast, G″ exhibited a steep increase before declining sharply, reflecting energy loss during deformation due to internal friction. This change signified a transition from gel-like to more fluid behavior due to network fragmentation and the loss of structural interactions. The crossover point, where G′ equals G″, marks the transition from solid to liquid, reinforcing the observations of Shah et al. (2016), who found that autoclaving and cooling reduced gelatinization enthalpy, highlighting the functional role of starch in various applications. Yield stress, or critical strain, obtained from strain sweep tests marks the transition from solid-like to flow behavior, indicating the onset of structural breakdown. Higher values reflected a stronger and more resilient gel network that better preserved texture during processing, whereas lower values suggested a weaker structure more prone to deformation. The yield stress values are included in the discussion of the strain sweep results.

The results demonstrated that autoclaving followed by storage at −10 °C significantly increased amylose content, promoted C to B-type crystallinity transition, and enhanced molecular ordering, leading to the highest RS content. The low-temperature induced structural rearrangements, particularly the formation of stable amylose double helices and strengthened hydrogen bonding, reduced enzymatic susceptibility and aligned with past findings in other starches. These retrogradation-driven changes also corresponded to reduced swelling, solubility, and peak viscosity, alongside higher setback viscosity and gel strength, indicating a more compact, resistant starch matrix.

The first stage of this study demonstrated that autoclaving followed by storage at −10 °C maximized RS yield (15.85 %) by promoting amylose double-helix formation, C to B-type crystallinity transition, and a compact, enzyme-resistant starch matrix. These retrogradation-driven changes also strengthened gels and reduced swelling, solubility, and peak viscosity. An autoclaved control without incubation was not included, but limiting separation of autoclaving versus storage effects was identified in Part 1 as optimal for RS enhancement, and was subsequently applied in Part 2 to glass noodle production. Freezing and cold incubation durations were optimized using RSM to preserve the texture while increasing RS, demonstrating a direct link between molecular structure modification and functional product performance.

### 3.2 Effect of incubation and freezing time on RS content, cooking quality, and texture properties of glass noodles

#### 3.2.1 Resistant starch content

**Fig. 3:**
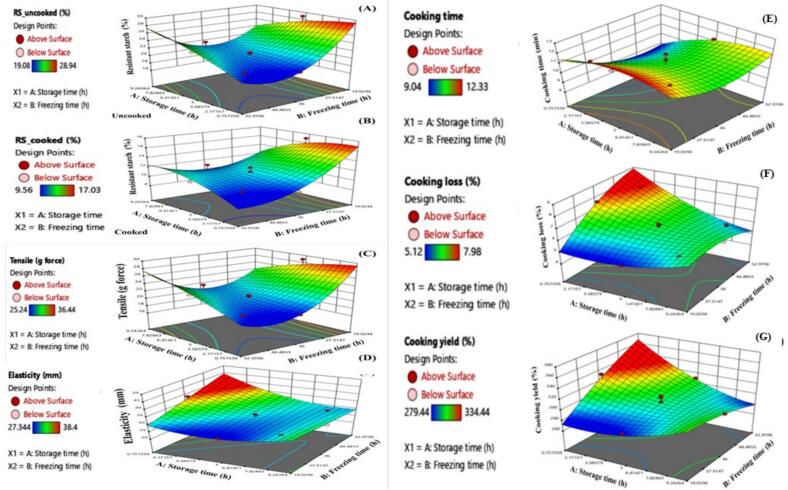
Response surface plots showing the effects of storage and frozen times on resistant starch (RS) contents of (A) uncooked, (B) cooked, (C) tensile strength (g force), (D) elasticity (mm), (E) cooking time (min), (F) cooking loss, and (G) cooking yield of glass noodles.

The RS content in uncooked glass noodles ranged from 19.08 % to 28.94 %, as shown in Table 2 and Fig. 3A. The freezing time showed a significant quadratic effect on RS content *(P ≤ 0.001)* while cold incubation time did not significantly affect RS levels (*P* > 0.05). Shorter freezing durations (19–25 h) initially reduced RS content, but levels subsequently increased, indicating a non-linear relationship driven by progressive starch retrogradation. Consistent with Part 1, autoclaving followed by low-temperature storage (−10 °C) promoted RS3 formation; however, total RS remained lower than in native starch due to the loss of RS2 from disrupted granules, outweighing the RS3 gain. Glass noodle production involves comparable heat treatments and the change from RS2 to RS3 is a key factor influencing both starch digestibility and the functional quality of the final product. These findings concurred with previous research on starchy foods such as rice, bread, and potatoes, where low-temperature incubation increased RS content, while reheating decreased it (Nguyen et al., 2023). After boiling the noodles for 10 min at 98–100 °C, RS content decreased to between 10.89 % and 17.03 %, as shown in Table 2 and Fig. 3B. The reduction in RS content observed after boiling was explained by extensive starch gelatinization, which disrupts the granular architecture and reduces RS1 and RS2 fractions. Starch leaching into the cooking water contributes to the overall loss of resistant starch, while cooling after boiling favors amylose retrogradation, promoting the formation of RS3. Thus, while cooking decreases the RS content due to gelatinization and leaching, subsequent cooling plays a crucial role in partially restoring RS levels through RS3 development. Freezing time (B2) exhibited a quadratic relationship with RS content, with linear correlations showing increased RS levels at longer freezing durations (*P ≤* 0.01). These results highlighted the significant role that freezing plays in modifying RS levels, due to retrogradation effects that occur during cooling. Optimal RS levels in glass noodles were achieved after two days of freezing. Cooking reduced RS content but retrogradation during the cooling process contributed to an increase in RS in the final product. These findings concurred with Nguyen et al. (2023), who reported that incubation at 4 °C for 1 h 30 min, followed by freezing at −10 °C for 18 h produced pure mung bean starch vermicelli with a high RS content and good quality. These results emphasized the importance of both freezing and cooling conditions in maximizing RS content in glass noodles and other starchy foods.

**Table 2:** Central composite design (CCD) showing the effect of the four independent variables on the seven dependent variables.

Run	Independent variables 1				Dependent variables						
	Coded level		Time (True values)		RS content (Y)		Cooking quality (Y)			Texture quality (Y)	
	X_1_	X_2_	A^2^ (h)	B^3^ (h)	Uncooked^4^ (%)	Cooked^5^ (%)	Time^6^ (min)	Loss^7^ (%)	Yield^8^ (%)	Tensile^9^ (g force)	Elasticity^10^ (mm)
1	1.41	0.00	9.30	36.00	21.51	10.89	10.64	6.23	302.33	30.44	30.44
2	0.00	−1.41	5.00	19.00	28.94	17.03	12.33	5.12	282.33	34.33	27.34
3	−1.00	−1.00	2.00	24.00	24.41	14.36	11.22	5.66	279.44	36.44	28.34
4	0.00	0.00	5.00	36.00	20.45	12.34	10.23	6.57	309.44	29.33	29.34
5	1.00	1.00	8.00	48.00	23.55	11.30	11.09	6.02	289.44	32.33	30.45
6	1.00	−1.00	8.00	24.00	21.34	11.02	11.76	6.12	299.33	28.34	30.44
7	0.00	0.00	5.00	36.00	21.34	11.23	10.33	6.67	312.34	30.98	31.33
8	0.00	0.00	5.00	36.00	21.12	11.34	11.76	6.09	286.46	29.44	27.45
9	0.00	0.00	5.00	36.00	20.12	10.34	11.22	6.02	286.44	29.56	28.45
10	0.00	0.00	5.00	36.00	20.12	11.44	10.20	6.57	300.44	30.56	31.555
11	−1.00	1.00	2.00	48.00	19.93	10.19	9.45	7.98	334.44	25.24	37.56
12	−1.41	0.00	0.00	36.00	19.08	9.56	9.04	7.58	332.34	26.34	38.40
13	0.00	1.41	5.00	53.00	23.83	11.12	11.23	6.34	310.46	28.34	32.35

1 Statistically significant difference.

2 A and X_1_, incubation time at 4 °C (h).

3 B and X_2,_ frozen time at −10 °C (h).

4 Y_1_ represents the yield (%) of RS of uncooked glass noodles.

5 Y_2_ represents the yield (%) of RS of cooked glass noodles.

6 Y_3_ represents the cooking time (min) of glass noodles.

7 Y_4_ represents the cooking loss (%) of glass noodles.

8 Y_5_ represents the cooking yield (%) of glass noodles.

9 Y_6_ represents the tensile (g force) of glass noodles.

10 Y_7_ represents the elasticity (mm) of glass noodles.

#### 3.2.2 Effect of incubation and freezing times on cooking quality

Our findings highlighted that incubation and freezing times significantly affected the cooking quality of glass noodles. Proper control of these conditions is essential for optimizing cooking time, cooking loss, and yield.

Cooking time: cooking times ranged from 9.04 to 12.33 min. Freezing time significantly affected cooking time, with longer freezing durations leading to increased cooking times (*P* ≤ 0.05), as shown in Table 2 and Fig. 3E. The observed quadratic relationship suggested that the effect of freezing on cooking time became more pronounced as freezing duration increased. This increase in cooking time was attributed to structural changes in the noodles that influenced heat absorption and water interaction. RS content, both in uncooked (*r* = 0.596) and cooked (*r* = 0.700) noodles, showed a positive correlation with cooking time, with longer cooking times associated with higher RS content. Cooking decreased the RS content due to starch gelatinization and leaching of soluble starch. Subsequent cooling promoted amylose retrogradation, forming RS3 and partially restoring RS levels. Therefore, the final RS content reflected the combined effects of cooking and cooling, with RS gains arising primarily from retrogradation during cooling rather than cooking duration alone. The positive correlation between RS content and cooking time suggested that longer cooking durations enhanced RS levels, particularly beneficial for specific dietary needs, as RS is linked to improved digestive health and may provide additional health benefits, such as better blood sugar control.

Cooking loss: cooking loss ranged from 5.12 to 7.98 %, as reported in Table 2 and Fig. 3F. A significant linear relationship (*P* ≤ 0.01, *P ≤* 0.01) was observed between incubation time and cooking loss, with longer incubation periods leading to reduced cooking loss, an important factor for noodle quality. High cooking loss resulted from the noodles dissolving in water, giving a cloudy solution with a sticky texture. Longer incubation and freezing times were associated with increased cooking loss, as indicated by the response surface graphs. Regression analysis revealed that extended incubation reduced cooking loss, which positively affected the final product. Ho Minh Thao and Noomhorm (2011) also reported that aging noodles at 4 °C showed reduced cooking loss and increased firmness through starch retrogradation. Excessive freezing (beyond 45 h) and prolonged incubation (7–9 h) led to increased cooking loss, due to ice crystal formation causing structural damage (Ribotta et al., 2003). The reduction in cooking loss with increased incubation suggested that retrogradation improved noodle firmness and reduced starch leaching during cooking. RS content showed a strong negative correlation with both cooking yield (*r* = −0.598) and cooking loss (*r* = −0.573), suggesting that as RS content increased cooking yield and starch loss during cooking decreased, reflecting a more stable noodle structure. The negative correlation between RS content and cooking yield and cooking loss indicated that higher RS content resulted in lower starch loss during cooking, leading to a more resilient noodle structure that better retained its form during cooking.

Cooking yield: cooking yield ranged from 279.44 to 334.44 %, as reported in Table 2 and Fig. 3G. Noodles with higher swelling power absorb more water, contributing to better texture and yield. Incubation time did not significantly affect cooking yield (*P* > 0.05), while freezing time (B) was associated with increased cooking loss. Incubation time negatively impacted cooking yield, while freezing time correlated with higher cooking loss but did not significantly affect yield (*P* > 0.05). Longer freezing times disrupted the noodle structure, with a balance needed to maintain quality. High cooking yield was linked to higher swelling power, which affects noodle texture (Seung-Young et al., 2005). Increased freezing time led to higher cooking loss, but did not significantly impact cooking yield. Cooking yield reflects the amount of water absorbed and retained by noodles, which directly influences their texture and eating quality. Higher cooking yield generally softens noodles but may also increase surface stickiness, whereas lower yield results in firmer, less sticky noodles that are preferred in certain cuisines.

#### 3.2.3 Effect of incubation and freezing times on the texture quality of glass noodles

The tensile strength of glass noodles ranged from 26.34 to 36.44 g force, as seen in Table 3 and Fig. 3C. Incubation at 4 °C significantly increased tensile strength (*P* ≤ 0.05), indicating that aging improved noodle hardness while reducing stickiness. This suggested that aging enhanced the structural integrity of glass noodles. Regression and ANOVA analyses showed that incubation time at 4 °C (A) significantly contributed to the increase in tensile strength (*P* ≤ 0.05). Lee et al. (2006) noted that aging noodles at 4 °C for 3–21 h increased hardness and reduced stickiness, due to enhanced tensile strength. Extended freezing at −10 °C negatively impacted tensile strength (*P* ≤ 0.001), highlighting that prolonged exposure to freezing conditions damaged the noodle structure. The interaction between incubation and freezing time (A*B) revealed that while tensile strength initially increased, prolonged freezing led to structural damage and reduced strength. The positive correlation between RS content and tensile strength suggested that higher RS levels contributed to stronger noodles, less prone to breaking. This observed enhancement in tensile strength with longer refrigeration suggested that temperature played a positive role in improving noodle texture and firmness. Conversely, extended freezing weakened the tensile strength, due to ice crystal formation disrupting the noodle matrix (Puhin et al., 2021). A significant positive correlation was observed between RS content and tensile strength uncooked (*r* = 0.781) and cooked (*r* = 0.712), indicating that higher RS content enhanced the structural integrity and firmness of the noodles. Texture Quality: the positive correlation with tensile strength indicated that higher RS content improved the firmness and structural integrity of the noodles.

**Table 3:** Analysis (ANOVA) of regression for the RS contents of uncooked and cooked glass noodles and cooking and texture properties of glass noodles.

Quadratic model	Lack of fit	R^2^	Final equation in terms of actual factors^1^, ^2^	Significant model^3^
Significant	Not significant	0.91	Y_1_ = 54.112 − 1.033 A − 1.641B +0.046AB − 0.047A^2^ + 0.018B^2^	A*, B*, AB*, A^2^ *, B^2^ ***
Significant	Not significant	0.84	Y_2_ = 31.059 − 0.388 A − 0.912B + 0.031AB − 0.074A^2^ + 0.009B^2^	B**, B^2^ *
Significant	Not Significant	0.80	Y_3_ = 16.285 + 0.395 A − 0.347B + 0.008AB − 0.049A^2^ + 0.004B^2^	A*, B*, B^2^ *
Significant	Not Significant	0.93	Y_4_ = 0.737 + 0.138 A + 0.273B − 0.017 A + 0.032A^2^ − 0.002B^2^	A**, B***, AB**, A^2^**, B^2^ **
Significant	Not significant	0.80	Y_5_ = 195.493 + 4.958 A + 4.570B − 0.451AB + 0.845A^2^ − 0.020B^2^	AB**, A^2^**
Significant	Not significant	0.91	Y_6_ = 59.900 − 2.919 A − 1.121B + 0.105AB − 0.068A^2^ + 0.006B^2^	B***, AB***
Significant	Not Significant	0.86	Y_7_ = 21.758 − 0.918 A + 0.489B − 0.064AB + 0.254A^2^ + 0.000011B^2^	A**, B**, A^2^**

1 Y_1_ and Y_2_ represent RS contents of uncooked and cooked glass noodles (%), Y_3_, Y_4_ and Y_5_ represent cooking time (min), cooking loss (%) and cooking yield (%) and Y_6_ and Y_7,_ represent tensile strength (g force) and elasticity (mm), respectively.

2 A, incubation time at 4 °C (h) and B_,_ frozen time at −10 °C (h).

3 *Significant at = *P* ≤ 0.05 level; ** Significant at = *P* ≤ 0.01, *P* ≤ 0.01 level; *** Significant at = *P* ≤ 0.001 level.

Elasticity values for the glass noodles ranged from 27.34 to 38.40 mm, as shown in Table 3 and Fig. 3D. The quadratic terms for incubation time (A^2^) showed a significant increase in elasticity (*P* ≤ 0.01) with incubation durations of 7–9.30 h, suggesting that optimal aging enhanced noodle texture. Kang et al. (2017) noted that non-frozen noodles were softer and stickier compared to noodles frozen for 24 h at −25 °C, aligning with our findings that incubation time positively affected elasticity. Elasticity significantly increased (*P* ≤ 0.01) with freezing times between 33 and 53 h, contributing to a desirable chewy and elastic texture. The negative correlation between RS content and elasticity indicated that as RS levels increased, the noodles became less stretchable, potentially affecting mouthfeel. The improvement in elasticity with longer freezing times suggested that freezing enhanced the textural properties of glass noodles, making them more appealing for consumers who prefer a chewy texture. RS content was also negatively correlated with elasticity uncooked: (*r* = −0.596), cooked: (*r* = −0.505), indicating that higher RS content reduced elasticity, which impacted chewiness and flexibility. The negative correlation with elasticity suggested that an increase in RS content reduced elasticity, potentially leading to a trade-off between firmness and chewiness. Higher RS promoted the formation of ordered crystalline regions during starch retrogradation, producing a denser, more rigid matrix (Kiumarsi et al., 2019). This structure enhances firmness and tensile strength but restricts starch chain mobility and gel network flexibility, lowering elasticity (Barros et al., 2018). By contrast, lower RS content results in a greater proportion of amorphous regions that retain more water and allow greater chain mobility, yielding a softer, more elastic texture. These findings revealed a clear RS–texture trade-off: higher RS improved firmness and structural stability, whereas lower RS enhanced chewiness. This mechanistic relationship between RS content, starch retrogradation, and contrasting effects on tensile strength and elasticity has rarely been reported in mung bean starch-based glass noodles, with this study one of the first to characterize and quantify these interactions. Understanding this balance is crucial for designing RS-enriched glass noodles that combine functional health benefits with consumer-preferred texture.

### 3.3 Regression for RS contents, cooking quality and texture properties of glass noodles

Quadratic models for the RS contents of uncooked and cooked glass noodles, along with cooking and texture properties, yielded regression coefficients that were highly significant (*P* ≤ 0.001). The coefficients of determination (R^2^) ranged from 0.80 to 0.93, demonstrating a strong predictive ability, as shown in Table 3. The 95 % confidence intervals confirmed that the RSM models explained a substantial portion of the variability in the data. The coefficient of variation (%CV) values ranged from 3.36 to 8.55 %, below the 10 % threshold, indicating the precision and reliability of the experimental design. The RSM approach was effective in characterizing the relationships between RS content and the cooking and texture properties of glass noodles, providing valuable insights for their production and quality.

The regression analysis (Table 1S) revealed that Y1 (RS content in uncooked noodles) decreased with increasing storage time at 4 °C (A) or frozen time at −10 °C (B), with curvature effects from quadratic terms. Y2 (RS content in cooked noodles) was more strongly influenced by B, with nonlinear effects from B^2^. Y3 (cooking time) increased with A but decreased with B, while B^2^ indicated nonlinear suppression. Y4 (cooking loss) increased with both A and B, with curvature and interaction effects from AB and quadratic terms. Y5 (cooking yield) was primarily driven by nonlinear effects of A^2^ and the AB interaction. Y6 (tensile strength) decreased with A and B individually, though the AB interaction partially mitigated this effect. Y7 (elasticity) decreased with A but increased with B and A^2^, indicating nonlinear and opposing influences.

The optimized −10 °C incubation of autoclaved mung bean starch, which promoted C to B-type crystallinity and enhanced RS3 content, translated effectively into glass noodle production. Freezing time (19–25 h) had the strongest impact on RS formation, while brief cold incubation (<5 h) fine-tuned texture, confirming that structural changes in starch governed functional noodle properties. The resulting noodles exhibited improved firmness, elasticity, and reduced digestibility, demonstrating the direct relationship between starch retrogradation, crystallinity, and RS enrichment, concurring with Nguyen et al. (2023). This study linked molecular structural changes to both RS content and functional performance, providing a practical approach for developing RS-enriched, low-glycemic glass noodles.

## 4 Conclusions

This study demonstrated that autoclaving combined with optimized sub-zero freezing and brief cold incubation, effectively enhanced RS content in mung bean starch-based glass noodles by inducing a change in crystallinity from C to B-type. These structural changes increased thermal stability, promoted RS3 formation, and reduced digestibility, offering potential health benefits for individuals with diabetes. Freezing time and cold incubation duration were critical in determining RS levels and noodle quality, with higher RS linked to reduced cooking yield and increased cooking loss. While this approach shows promise for developing low-glycemic, functional glass noodles, future research should address sensory acceptance, shelf-life stability, industrial-scale feasibility, and in vivo health impacts, including a glycemic response and gut microbiota modulation.

## CRediT authorship contribution statement

**Ratchaneeporn Photinam:** Writing – original draft, Visualization, Validation, Software, Methodology, Investigation, Formal analysis, Data curation, Conceptualization. **Pakkawat Detchewa:** Writing – original draft, Validation, Supervision, Resources, Methodology. **Anuchita Moongngarm:** Writing – review & editing, Writing – original draft, Supervision, Resources, Project administration, Funding acquisition, Conceptualization.

## Declaration of generative AI and AI-assisted technologies in the writing process

ChatGPT was used to correct the English grammar. After using this tool/service, the authors reviewed and edited the text, and take full responsibility for the published content.

## Declaration of competing interest

The authors declare that they have no known competing financial interests or personal relationships that could have appeared to influence the work reported in this paper.

## Data Availability

Data will be made available on request.
