# Supplementary material for: Enhancing resistant starch and quality of mung bean starch and glass noodles through optimized incubation times and temperatures
Source: Food Chem X. 2025 Oct 1;31:103111. doi: 10.1016/j.fochx.2025.103111 (PMC12528911; doi:10.1016/j.fochx.2025.103111)
Supplement: Supplementary material [file mmc1.docx]

**Standard curve of amylose (Fig. S1) and the regression analysis (Table 1S)**


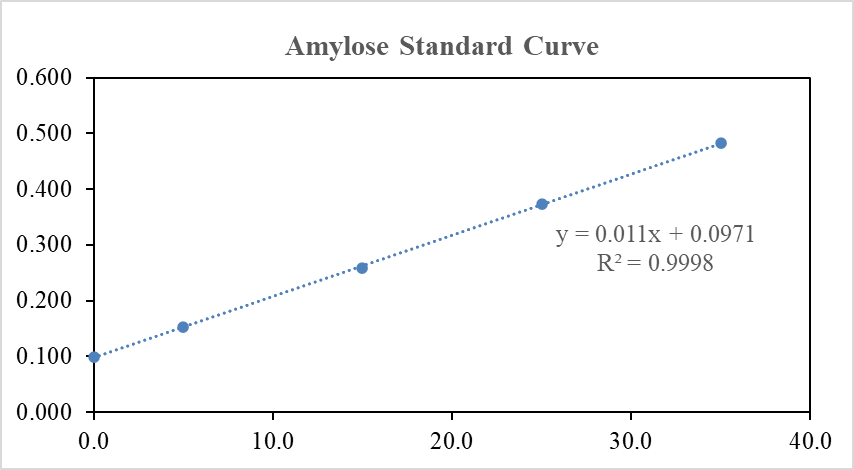

Fig. S1. Standard curve used to determine the amylose content in mung bean starch.

Table S1. Regression coefficients and statistical significance for the key response variables

| Response^1^ | Intercept | A (linear)^2^ | B (linear)^2^ | AB (interaction) | A² (quadratic) | B² (quadratic) | R² | Key interpretations |
| --- | --- | --- | --- | --- | --- | --- | --- | --- |
| Y1 | 54.112 | –1.033* | –1.641* | +0.046* | –0.047* | +0.018*** | 0.91 | Increasing A or B reduces Y1; curvature observed from quadratic terms |
| Y2 | 31.059 | –0.388 | –0.912** | +0.031 | –0.074 | +0.009* | 0.84 | Y2 is more sensitive to B; nonlinear effects from B² |
| Y3 | 16.285 | +0.395* | –0.347* | +0.008 | –0.049 | +0.004* | 0.80 | A increases Y3, B decreases it; B² shows nonlinear suppression |
| Y4 | 0.737 | +0.138** | +0.273*** | –0.017** | +0.032** | –0.002** | 0.93 | Both A and B enhance Y4; AB and quadratic terms indicate curvature and interaction |
| Y5 | 195.493 | +4.958 | +4.570 | –0.451** | +0.845** | –0.020 | 0.80 | Nonlinear effect of A² and AB interaction are primary drivers |
| Y6 | 59.900 | –2.919 | –1.121*** | +0.105*** | –0.068 | +0.006 | 0.91 | A and B decrease Y6 individually; AB interaction partially offsets this |
| Y7 | 21.758 | –0.918** | +0.489** | –0.064 | +0.254** | +0.00001 | 0.86 | Reduced by A, but increased by B and A², indicating nonlinear and opposing influences. |

^1^ Y_1_ and Y_2_ represent RS contents of uncooked and cooked glass noodles (%), Y_3_, Y_4_ and Y_5_ represent cooking time (min), cooking loss (%) and cooking yield (%) and Y_6_ and Y_7,_ represent tensile (g force) and elasticity (mm), respectively.
^2^ A, storage time at 4°C (h) and B_,_ frozen time at -10 °C (h).
*Note: *, *, *** indicate significance at *P* < 0.05, *P* < 0.01, and *P* < 0.001, respectively.
